# Huge Fibroid in Pregnancy: A Case Presentation

**DOI:** 10.7759/cureus.59566

**Published:** 2024-05-03

**Authors:** Athanasios Petroulakis, Emmanouil Katsanevakis, Bing Tiong, Sajida Ajjawi

**Affiliations:** 1 Obstetrics and Gynaecology, Nottingham University Hospitals NHS Trust, Nottingham, GBR

**Keywords:** antenatal scan, radiology interventional, huge fibroid, uterine fibroid in pregnancy, gestation age, caesarean section

## Abstract

Uterine fibroid, widely known as leiomyoma, is one of the most common benign tumours of the female reproductive system. It is not uncommon for pregnancies to be complicated by uterine fibroids. Most commonly, the first line of large uterine fibroids management in pregnancy is conservative, with myomectomy counselling after delivery if necessary. In this paper, we present a case of a very high-risk pregnancy, that was managed by delivery via caesarean section at 34 weeks of gestation, which was performed for a patient, with an 18 centimetres (cm) fibroid, first diagnosed during pregnancy. Interventional radiology involvement was critical in this case for minimizing the final blood loss and surgical complications. Bilateral internal iliac artery balloons were used. Maternal and foetal risks, the timing of delivery, and the options for the management of fibroids in pregnancy will be discussed.

## Introduction

The prevalence of uterine fibroids is approximately 20%-60% of women of reproductive age. The incidence of leiomyomas in pregnancy may be underestimated since the fibroids can very commonly be asymptomatic [1]. The prevalence of fibroids found in pregnant women has risen as more women prefer to postpone starting a family until a later stage of their lives [2]. Pregnancy-related hormones can influence the size of uterine leiomyomas. However, there is insufficient research data to suggest if fibroids grow in pregnancy. Patients with uterine fibroids in pregnancy need to be extensively counselled about the possible adverse outcomes and risks. However, most women with fibroids have uneventful pregnancies [3]. Despite the growing prevalence, the relationship between uterine fibroids and adverse pregnancy outcomes is not clearly understood. Miscarriage, premature labour, antepartum haemorrhage, malposition, malpresentation, obstructed labour, uterine inversion, postpartum haemorrhage, and puerperal sepsis are among the possible obstetric consequences of co-existing uterine fibroids in pregnancy [4]. Advances in radiology and, more specifically, in ultrasound have led to improvement in the diagnosis of uterine fibroids in pregnancy. According to the literature, antepartum and caesarean myomectomies have been performed successfully in selected cases [1]. This paper aims to present a very interesting antenatal management and delivery plan of a pregnant patient who was undiagnosed as having an 18cm uterine fibroid.

## Case presentation

A 30-year-old nulliparous female, presented for her first trimester trans-vaginal nuchal translucency (NT) scan, at 12 weeks of gestation. As per the scan report, the uterus appeared bulky and coarse and contained multiple, ill-defined fibroids. The endometrium could not be visualised, and no gestational sac was seen. A well-circumscribed, heterogenous mass was identified, arising from the midline, and extending to both adnexa, measuring 51x48x45 mm, with strong vascular flow. These appearances were thought to be suggestive of an ectopic pregnancy. The patient was urgently referred to the emergency gynaecology triage unit (GTU) for further management of a possible ectopic pregnancy. As the patient arrived at the GTU, the clinical examination was suggestive of a fibroid uterus. There was no tenderness elicited during the examination. The plan from the gynaecologist was to perform a repeat β-Chorionic Gonadotropin (β-hCG) in 48hrs, and an urgent scan was arranged.

**Figure 1:**
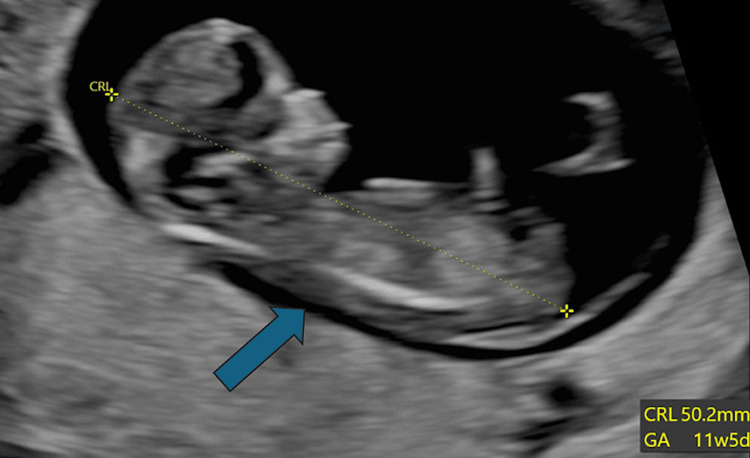
Transvaginal scan: CRL and foetus seen, one day after the initial presentation.

**Figure 2:**
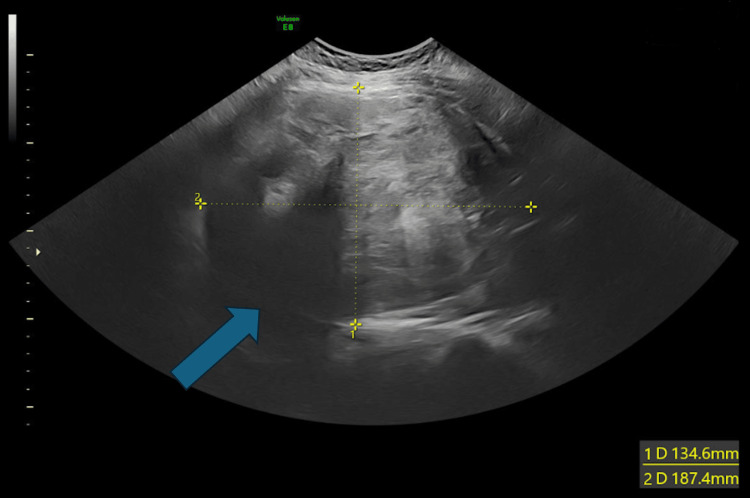
Transvaginal scan: a 187*134mm uterine fibroid was seen.

A further scan was performed, and an intrauterine pregnancy was eventually confirmed. The fetus was localised, and the crown-rump-length (CRL) measured 50.2mm, which is equivalent to a gestation age (GA) of 11 weeks and five days (Figure 1). A large fibroid measuring 187mm was identified (Figure 2), along with a 5cm cervical fibroid. The large fibroid and the foetus were both not seen during the antenatal clinic scan appointment (A scan), possibly due to distortion of the anatomy and shadowing of the cervical fibroid, which was initially thought to be an ectopic pregnancy.

 

**Figure 3:**
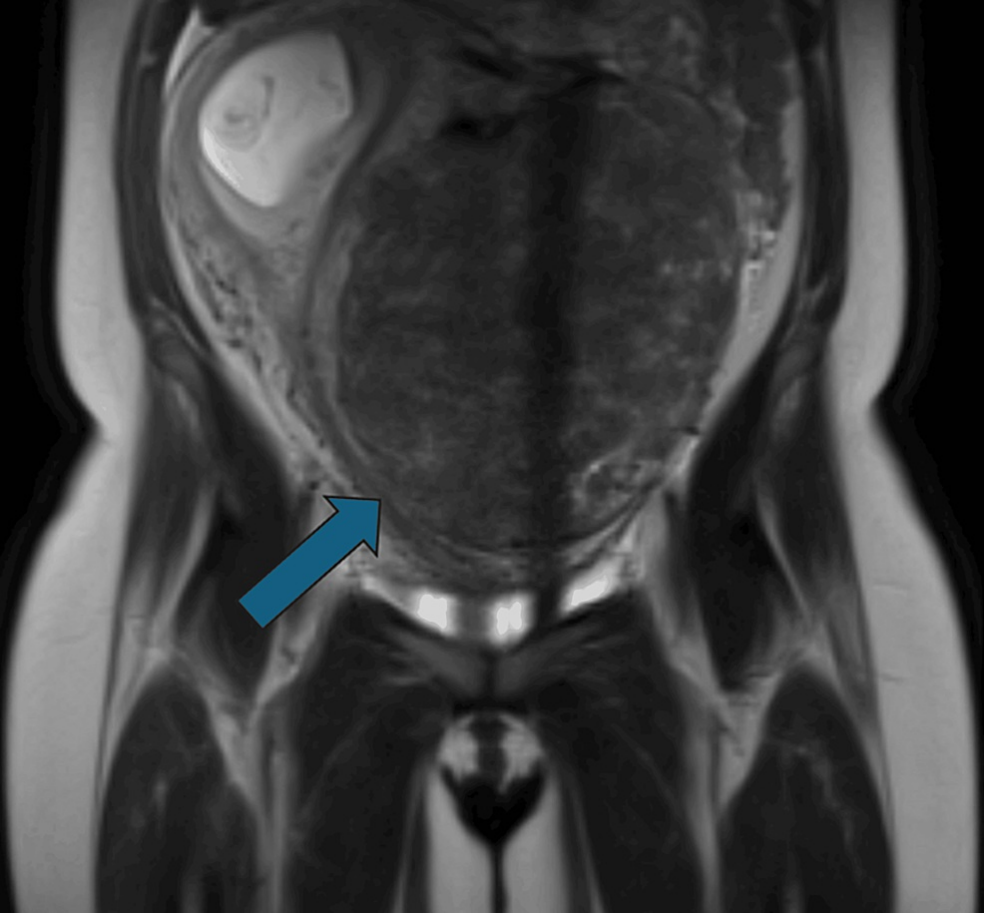
Anterior view of the MRI scan (abdomen and pelvis). The fibroid is seen.

**Figure 4:**
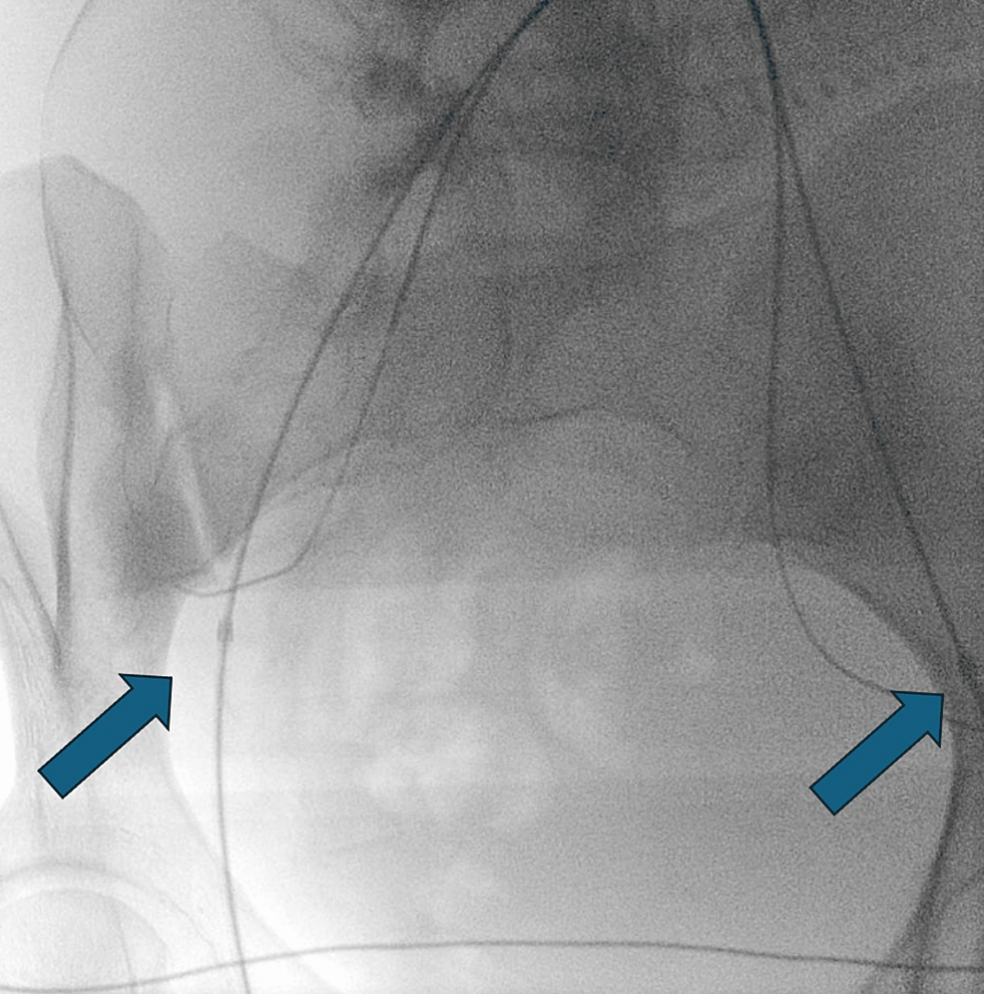
Bilateral internal iliac artery balloons.

An MRI scan was requested to further assess the fibroid uterus and its anatomical relations, six days after the initial presentation. The uterus was in a neutral position with a typical intramural fibroid measuring 181*130*158 mm, displacing the endometrial cavity to the right (Figures 3, 4). The cavity contained the placenta and fetus and there were no features suggesting possible cornual or angular pregnancy. There was also a 70*50 mm mass in the pouch of Douglas with fibroid texture. This is the mass that was visualized at the initial first trimester scan appointment. Left-sided hydro-nephrosis and hydroureter were evident, most likely due to compression effects from the fibroid. The urology team advised that the patient was not a candidate for ureteric stent at the time since the kidney function was normal and the pressure changes was unilateral. The patient was counselled on the option of termination of pregnancy, the high risk of miscarriage, preterm birth, bleeding risks and hysterectomy. The patient decided to continue with the pregnancy. 

Early multidisciplinary team (MDT) meeting input was sought, considering the benefits and risks of the ongoing pregnancy with a massive uterine fibroid versus the high risk of miscarriage and bleeding, and the challenges of an emergency caesarean section. One option discussed with the patient taking considering those risks, was a planned elective Caesarean section at 34 weeks. The alternative option was to adopt a watchful waiting period after 34 weeks, with risks surrounding the need for an emergency section. After counselling, patient preferred to have an elective Caesarean section at 34 weeks of gestation. The decision for an elective caesarean section at 34 weeks was made, with input from the interventional radiology and experienced anaesthetist, as well as the presence of experienced gynaecology consultants. The woman was started on iron tablets at 25 weeks of gestation as precaution and to optimise haemoglobin level prior to delivery. A course of antenatal corticosteroids was also given at 29+3 and 29+4 weeks of gestation.

On the day of the caesarean section, interventional radiology team performed a bilateral internal iliac artery balloon occlusion to reduce the risk of bleeding (Figure 4). Six units of red blood cells were cross-matched prior to the operation. An ultrasound scan was performed by an experienced consultant, to assist in the decision-making regarding the type of uterine incision that would be performed. A midline skin incision and routine entry into the peritoneal cavity was performed. The large fibroid uterus was evident, and the biggest fibroid distorted the normal female pelvis anatomy and uterine angles. A lateral-transverse incision was considered the best approach, to avoid major vessels and fibroid tissue. The foetus was successfully delivered in breech presentation without concerns. Immediately after delivery, the balloons were inflated bilaterally with normal saline. Once the uterus was completely repaired in two-layers using 2-0 Vicryl suture and the blood loss was controlled, the balloons were deflated. The total measured blood loss was 2,300 millilitres (mL). Uterotonics were used to prevent further blood loss. Ten international units (IU) of oxytocin were given, as well as 2 grams (gr) of tranexamic acid intravenously (IV) and 250 micrograms (mcg) of Carboprostol. Cell salvage was used, and 400 ml of patient’s own blood was transfused back after the operation.

A baby girl weighed 2020g was born in good condition with APGAR score of 3, 6, and 7 at 1/5/10 minutes of age, respectively. The woman was discharge on day 3 post operation. She made a full recovery from the operation, with no complication. There was no further blood products transfusion post-surgery. She was reviewed in outpatient clinic four months post-delivery and there was no concern raised or identified. Follow-up ultrasound scan showed a similar findings of fibroids uterus.

## Discussion

This is a case of a very high-risk pregnancy due to the incidental sonographic finding of a huge uterine fibroid in the first trimester of pregnancy. Understanding the increased risks of continuing the pregnancy and counselling the patient about the material risks are crucial. The temporal association of the delivery plan is challenging. Early MDT involvement is crucial. MDT management to reduce the risk of bleeding by involving interventional radiology, as well as the precise timing of the delivery by balancing the risks and the foetal growth are the main points of this case presentation.

Most diagnoses of big uterine fibroids during pregnancy are made in the first trimester, as more pregnant patients start their antenatal care in the first trimester, which makes it easier to establish the diagnosis [3]. However, as shown in our case, the diagnosis of fibroids in pregnancy is not always straightforward and can be challenging. Many authors have commented on the challenges of uterine fibroid diagnosis in pregnancy and the limitations of ultrasound in differentiating fibroid tissues from the physiological myometrial thickening in pregnancy [5,6]. The detection and evaluation of fibroids as the pregnancy progresses is difficult, due to the changes in uterine anatomy and the presence of the foetus and placenta, that sometimes make the examination difficult and often painful for the patient. Therefore, it is recommended to perform all necessary imaging studies in the first trimester to aid a multidisciplinary management plan for antenatal, intrapartum, and postpartum care. 

Most fibroids are asymptomatic. Severe abdominal pain can occur if the fibroids undergo red degeneration or torsion (for example with a pedunculated subserosal fibroid) [7]. Red degeneration happens when rapid fibroid growth results in the tissue outgrowing its blood supply leading to tissue necrosis and infarction. Three theories have been proposed in order to explain the pain associated with red degeneration: a) The growing uterus in pregnancy results in a change in the architecture of the vascular supply to the fibroid leading to ischaemia and necrosis [8], b) the rapid fibroid growth leads to inadequate blood supply towards it, which in turn leads to anoxia and infarction [9] and c) destruction of cells within the fibroid leads to prostaglandin release and pain [10]. The main effect of fibroids on pregnancy is related to the size of the uterine fibroids [11].

Uterine fibroids are connected with an increased risk of early pregnancy loss. Fibroids developed in the uterine body are more likely to cause miscarriage compared to fibroids in the lower uterine area [12]. Factors such as increased uterine irritability and contractility due to uterine fibroids can lead to increased pregnancy loss, likely related to the compressive effect and disruption of blood flow to the placenta and foetus. This is more likely when placenta implants close to a fibroid nodule [2]. 

The risks of placenta praevia and placenta abruption are increased in pregnant women with fibroid uterus. Submucosal fibroids, retroplacental fibroids, and fibroid volume > 200 centimetres (cm) are risk factors for placental abruption. [13] A possible explanation for this could be the diminished blood flow to the fibroid and the adjacent tissues which results in partial ischaemia and decidual necrosis in the placental tissues overlying the fibroids [14]. Pregnant women with uterine fibroids are more likely to go into preterm labour compared to women without fibroids, and this is more prevalent in those with multiple fibroids and in those with fibroids contacting the placenta [12]. Labour and delivery are more difficult in the case of foetal malpresentation and malposition, which can be due to uterine distortions produced by fibroids. Large fibroids, multiple fibroids, and fibroids in the lower uterine segment are risk factors for malpresentation [13]. Patients with uterine fibroids are more likely to have a retained placenta. In addition, they tend to have dysfunctional labours because of the distortion of the uterine architecture and the interference of myometrial contractility, and as a result, they are more likely to utilize uterotonics to control uterine contractions and to ensure that the labour advances properly. 

After the baby and placenta are delivered, this effect on myometrial contractility repeats spontaneously, resulting in uterine atony and postpartum haemorrhage [1]. Myomectomies during caesarean section may merely extend the surgical duration and prolong the postoperative hospital stays. Myomectomies during caesarean section is known to cause significant haemorrhaging because the pregnant uterus has increased vascularity. Therefore, it is commonly suggested that myomectomies be avoided as much as possible during caesarean delivery. The use of oxytocin and interventional radiology to prevent postpartum haemorrhaging is recommended in the cases of parturients with uterine fibroids [15]. 

MDT involvement is crucial in managing complex cases such as pregnancies complicated by huge fibroids. MDT brings together experts from various specialties like obstetrics, gynaecology, radiology, and anaesthesia. Each specialist contributes unique insights to comprehensively assess the situation, considering both the maternal and foetal health implications. MDT helps to tailor management plans to individual patient needs. In our case of huge fibroids in pregnancy, the team discussed various treatment options ranging from conservative management to surgical interventions, weighing the risks and benefits for both the mother and the baby. MDT involvement ensures that the material risks are identified early, and appropriate measures are taken to mitigate them. For instance, the team decided on closer monitoring, timely interventions, or also an elective caesarean section to minimise risks. MDT meetings provide a platform for shared decision-making, where the women can discuss preferences, concerns, and expectations with a diverse team of experts. This collaborative approach fosters trust and ensures that the chosen management plan aligns with the women's goals and values. MDT involvement in obstetric care improves clinical outcomes, including reduced maternal and neonatal morbidity and mortality rates. By harnessing the collective expertise of various specialties, MDT can optimise care delivery and improve patient outcomes in complex cases like pregnancies complicated by huge fibroids. In essence, MDT involvement ensures that pregnant women receive holistic, evidence-based care that addresses the complexities of their condition while prioritising their well-being and the health of their baby.

## Conclusions

Fibroids are a common uterine pathology, and many women may have them without experiencing any symptoms or problems during pregnancy. Ultimately, many women with fibroids go on to have healthy pregnancies and deliveries, but close medical supervision is essential to address complications that may arise, as a distinct cohort may confront major risks, including pregnancy loss, preterm birth, placenta abruption, and major obstetric haemorrhage. The management of uterine fibroids in pregnancy is a multifaceted endeavour, that necessitates vigilant oversight and strategic, multidisciplinary intervention.

Imaging to evaluate the exact anatomical relations of the fibroid and adjacent structures is crucial. Interventional radiology and the use of cell salvage are both helpful parameters to control blood loss and facilitate autologous transfusion. The decision on the type of uterine incision is also vital. It is noteworthy for women with uterine fibroids who are planning to become pregnant or are already pregnant to discuss the risks and prognosis with their healthcare provider in advance. Thus, personalized care, based on the specific characteristics of the fibroids and the individual's health should be provided. In our case, the approach of the timing of delivery by a Caesarean section at 34 weeks, in view of the increased risk of bleeding and antenatal as well as intrapartum adverse outcomes, finally led to safe delivery of a well-grown baby and minimal surgical adverse outcomes.

## References

[1] Eyong E, Okon OA (2020). Large uterine fibroids in pregnancy with successful Caesarean myomectomy. Case Rep Obstet Gynecol.

[2] Egbe TO, Badjang TG, Tchounzou R, Egbe EN, Ngowe MN (2018). Uterine fibroids in pregnancy: prevalence, clinical presentation, associated factors and outcomes at the Limbe and Buea Regional Hospitals, Cameroon: a cross-sectional study. BMC Res Notes.

[3] Pavone D, Clemenza S, Sorbi F, Fambrini M, Petraglia F (2018). Epidemiology and risk factors of uterine fibroids. Best Pract Res Clin Obstet Gynaecol.

[4] Cerdeira AS, Tome M, Lim L (2021). The value of MRI in management of uterine fibroids in pregnancy. Eur J Obstet Gynecol Reprod Biol.

[5] Qidwai GI, Caughey AB, Jacoby AF (2006). Obstetric outcomes in women with sonographically identified uterine leiomyomata. Obstet Gynecol.

[6] Cooper NP, Okolo S (2005). Fibroids in pregnancy--common but poorly understood. Obstet Gynecol Surv.

[7] Lee HJ, Norwitz ER, Shaw J (2010). Contemporary management of fibroids in pregnancy. Rev Obstet Gynecol.

[8] Parker WH (2007). Etiology, symptomatology, and diagnosis of uterine myomas. Fertil Steril.

[9] De Carolis S, Fatigante G, Ferrazzani S, Trivellini C, De Santis L, Mancuso S, Caruso A (2001). Uterine myomectomy in pregnant women. Fetal Diagn Ther.

[10] Katz VL, Dotters DJ, Droegemeuller W (1989). Complications of uterine leiomyomas in pregnancy. Obstet Gynecol.

[11] Benson CB, Chow JS, Chang-Lee W, Hill JA 3rd, Doubilet PM (2001). Outcome of pregnancies in women with uterine leiomyomas identified by sonography in the first trimester. J Clin Ultrasound.

[12] Lev-Toaff AS, Coleman BG, Arger PH, Mintz MC, Arenson RL, Toaff ME (1987). Leiomyomas in pregnancy: sonographic study. Radiology.

[13] Vergani P, Locatelli A, Ghidini A, Andreani M, Sala F, Pezzullo JC (2007). Large uterine leiomyomata and risk of cesarean delivery. Obstet Gynecol.

[14] Exacoustòs C, Rosati P (1993). Ultrasound diagnosis of uterine myomas and complications in pregnancy. Obstet Gynecol.

[15] Brown D, Fletcher HM, Myrie MO, Reid M (1999). Caesarean myomectomy--a safe procedure. A retrospective case controlled study. J Obstet Gynaecol.

